# Critical factors for flow cytometry analysis of the brain

**DOI:** 10.1111/febs.70063

**Published:** 2025-03-14

**Authors:** Mizuki Sadakata, Ayumu Konno, Akinori Takase, Tetsuhiro Kasamatsu, Takatoshi Iijima, Hirokazu Hirai, Tetsushi Sadakata

**Affiliations:** ^1^ Education and Research Support Center Gunma University Graduate School of Medicine Maebashi Gunma Japan; ^2^ Department of Neurophysiology and Neural Repair Gunma University Graduate School of Medicine Maebashi Gunma Japan; ^3^ Viral Vector Core Gunma University Initiative for Advanced Research (GIAR) Maebashi Gunma Japan; ^4^ Department of Life Science Support, Research Innovation Center University Hospitals Sector, Tokai University Isehara Kanagawa Japan; ^5^ Department of Medical Technology and Clinical Engineering Gunma University of Health and Welfare Maebashi Gunma Japan; ^6^ Department of Molecular Life Science, Division of Basic Medical Science and Molecular Medicine, School of Medicine Tokai University Isehara Kanagawa Japan

**Keywords:** autofluorescence, brain, exosome, flow cytometry

## Abstract

The brain is difficult to analyze using flow cytometry due to its complex interactions with cells, high lipid content, and high autofluorescence. In this study, we investigated methods to isolate various types of brain cells with high yield and viability. The results showed that protease selection significantly affected the viability of various cell types in the brain. Differences in the developmental stage also affected cell yield and viability. Furthermore, the intensity of autofluorescence differs greatly between various regions of the brain. Additionally, we searched for neuronal indicators capable of identifying a diverse range of neurons. The ratios of various exosomes contained in neurons differ depending on the type of neuronal marker. These results revealed critical factors that must be considered when analyzing various types of brain cells using flow cytometry.

## Graphical abstract

The brain is difficult to analyze using flow cytometry due to its cellular complexity, high lipid content and high autofluorescence. This study explored methods to isolate brain cells for flow cytometry, revealing that protease selection and developmental stage significantly affect cell viability and yield. Autofluorescence varies across brain regions, and neuron markers differ in identifying neuron types. These results showed critical factors that must be considered when analyzing various types of brain cells using flow cytometry.

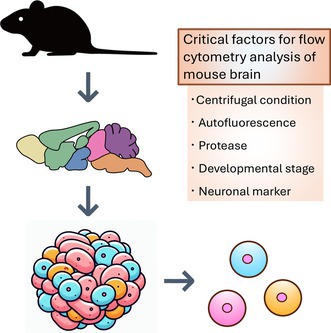

**Abbreviations**
7‐AAD: 7‐amino‐actinomycin D
AAV: adeno‐associated virus
APC: allophycocyanin
CNS: central nervous system
DAPI: 4′,6‐diamidino‐2‐phenylin‐dole
FBS: fetal bovine serum
FSC: forward scatter
GFP: green fluorescent protein
GLAST: glutamate aspartate transporter
HBSS: Hanks' balanced salt solution
MAP2ab: microtubule‐associated protein 2a + 2b
NCAM: neural cell adhesion molecule
NSE: neuron‐specific enolase
PE: phycoerythrin
PerCP: peridinin chlorophyll protein
scRNA‐seq: single‐cell RNA sequencing
SIP: solution of isotonic Percoll
SSC: side scatter
VSMC: vascular smooth muscle cell

## Introduction

Western blot and immunohistochemistry are commonly used to analyze the brain. However, each method has limitations. For example, western blot performed on homogenized brain samples does not determine the type of brain cells in which the subunits are expressed or their subcellular locations (membrane or intracytoplasmic). Immunohistochemistry is a common method used to visualize specific cells and proteins in the brain, and recent algorithms have improved the semiquantification of fluorescence intensity [1, 2]. However, the entire procedure is time‐consuming and not suited to processing large numbers of samples.

Flow cytometry is a highly quantitative method with numerous applications in basic and clinical research [3]. Using flow cytometry, samples can be prepared and fixed in hours; these fixed samples can then be stored, stained, and analyzed. Staining the samples takes only several hours for a large number of samples. Running the samples takes several minutes per sample; thus, the flow cytometry protocol can be carried out from the animal sacrifice to completion in half a day [4]. Therefore, the principal advantages of flow cytometry over immunohistochemistry‐based approaches are the speed and relative ease with which the assay is performed.

This technique allows simultaneous characterization and quantification of multiple phenotypic and functional parameters within a single cell or cell population. Semiquantitative parameters such as particle size (forward scatter, FSC) and granularity (side scatter, SSC) can easily be determined [5]. Labeling cells or particles with fluorescent antibodies and/or dyes enables the identification of cells or particles in complex solutions.

However, measuring brain tissue using flow cytometry is difficult due to the complex and intricate structure of the brain [6]. Furthermore, the brain is composed of myelin, which is rich in lipids and exhibits high autofluorescence [7]. These characteristics make it challenging to analyze brain tissue using flow cytometry.

This study aimed to develop a reproducible, quantitative flow cytometric method for neuronal and non‐neuronal cell characterization that minimizes the errors associated with traditional semiquantitative stereological techniques, thus increasing the throughput and reliability of the acquired data obtained within a given sample. Neuroscience will benefit from the optimal use of flow cytometry to elucidate physiological and pathological processes.

## Results

### Removal of myelin debris during preparation of brain cells

**Fig. 1:**
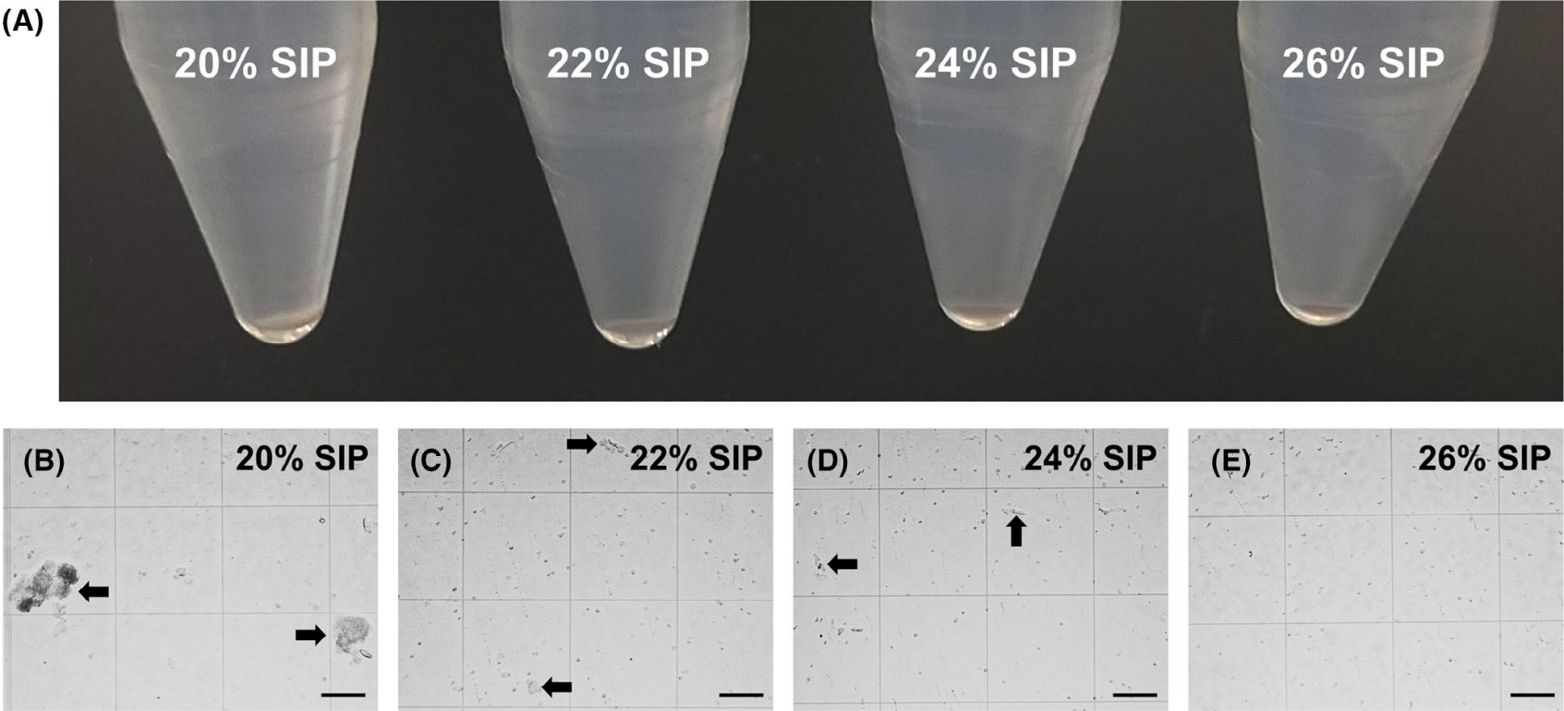
Removal of myelin debris with different concentrations of SIP. (A) Precipitate after centrifuging the lysate of P35 mouse whole brain after papain treatment at 20–26% solution of isotonic Percoll (SIP). The white precipitate was myelin debris. The reproducibility of the data was confirmed three times, and a representative result is shown. (B–E) Precipitates after centrifugation at 20 (B), 22 (C), 24 (D), and 26% SIP (E) were observed using a hemocytometer. Arrows indicate myelin debris. Scale bars, 100 μm. The reproducibility of the data was confirmed three times, and a representative result is shown.

Generally, 30–37% Percoll is used to remove myelin and cell debris from the brain and spinal cord and to collect microglia and leukocytes [4, 8, 9, 10, 11, 12]. We attempted centrifugation with a smaller concentration of Percoll to separate and collect more brain cells from myelin debris. When centrifuged in 20% stock isotonic Percoll (SIP), myelin precipitated at the bottom of the tube along with the cells (Fig. 1A). When centrifuged at ≥22% SIP, white myelin precipitates were not observed (Fig. 1A). However, the myelin debris was scattered with 20% (Fig. 1B) and 22% SIP (Fig. 1C) when the pellets were observed using a hemocytometer. Only a small amount of myelin debris was observed with 24% SIP (Fig. 1D), while no myelin debris was observed with 26% SIP (Fig. 1E). Based on these results, cells were centrifuged at 24% SIP for subsequent experiments.

### Differences in autofluorescence in brain regions

**Fig. 2:**
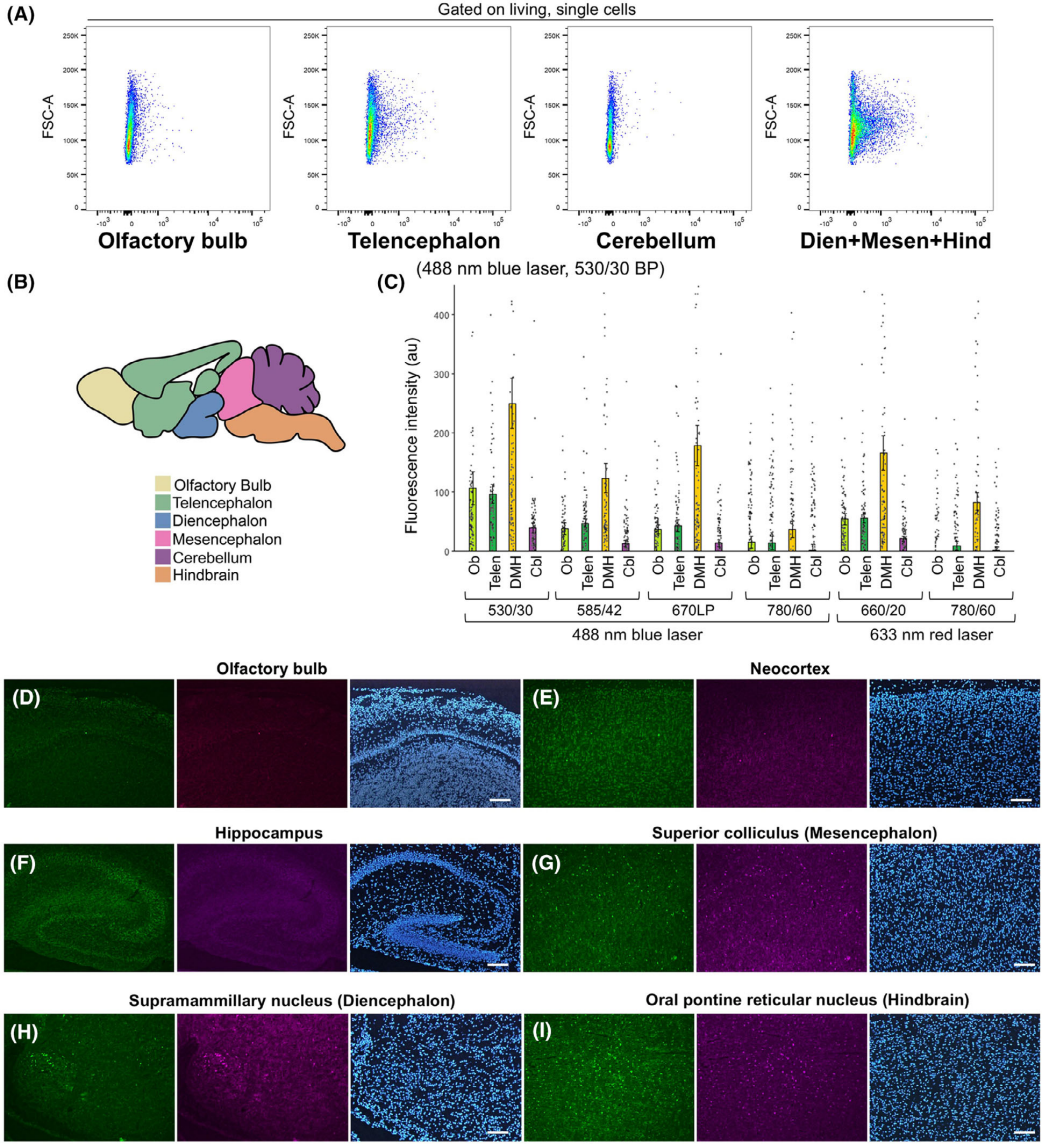
Differences in autofluorescence in mouse brain regions. (A) Flow cytometry data showing the autofluorescence of brain cells prepared from the four regions. A 488‐nm blue laser was used to detect the autofluorescence signal with a 530/30 nm bandpass filter. Dien+Mesen+Hind indicates brain cells prepared from the diencephalon, mesencephalon, and hindbrain. The papain‐treated P8 mouse brain was used in this study. (B) Illustration of the brain regions used in this study. (C) The intensity of autofluorescence of cells obtained from four regions of the brain (*n* = 100 for each bar). Cbl, cerebellum; DMH, Dien+Mesen+Hind; Ob, olfactory bulb; Telen, telencephalon. Error bars represent mean ± SEM. (D–I) Green (460–495 nm excitation and 510–550 nm emission) and magenta (543/22 nm excitation and 593/40 nm emission) autofluorescence and 4′,6‐diamidino‐2‐phenylindole (DAPI) (blue) of unstained sagittal sections of C57BL/6J mice at P8. Scale bars, 100 μm. Reproducibility was confirmed with sections made from three different mice, and a representative result is shown.

We examined the relationship between differences in the brain region and autofluorescence intensity in isolated cells. The brain was divided into four regions: the olfactory bulb, telencephalon, cerebellum, and other brain regions (diencephalon, mesencephalon, and hindbrain) (Fig. 2B). The fluorescence intensity of the unlabeled cells was measured using flow cytometry (Fig. 2A). The brain regions, including the diencephalon, mesencephalon, and hindbrain, showed high autofluorescence with any fluorescent laser or filter set (Fig. 2C). The olfactory bulb and telencephalon cells showed slightly higher autofluorescence when observed using a 488 nm blue laser and a 530/30 BP filter (Fig. 2A,C).

Consistent with this, stronger green (460–495 nm excitation and 510–550 nm emission) and red (543/22 nm excitation and 593/40 nm emission) autofluorescence was observed in the mesencephalon (Fig. 2G), diencephalon (Fig. 2H), and hindbrain (Fig. 2I) than in the olfactory bulb (Fig. 2D), neocortex (Fig. 2E), and hippocampus (Fig. 2F) when unstained tissue was observed under a fluorescence microscope.

### Validation of markers that recognize neurons in flow cytometry

**Fig. 3:**
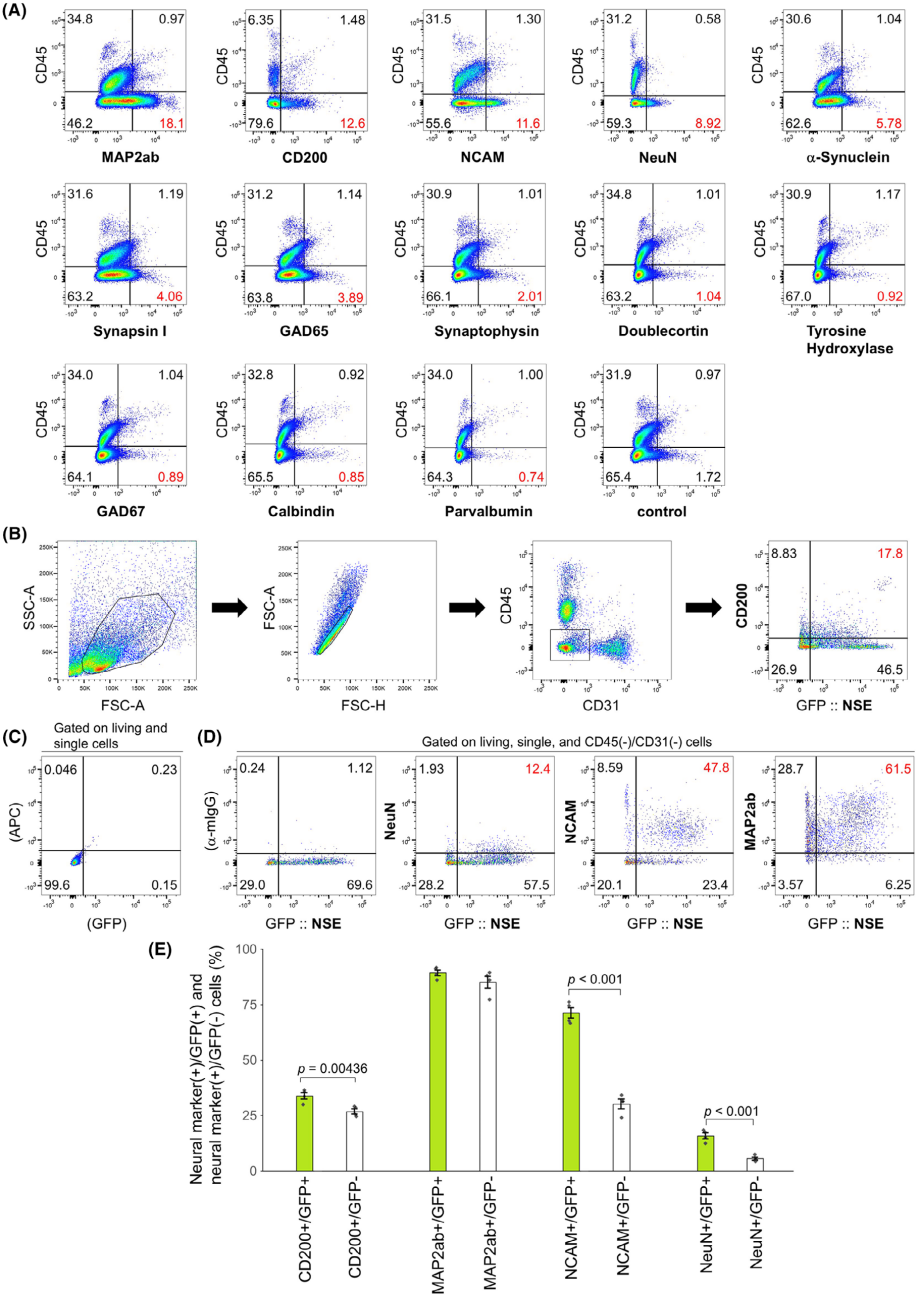
Validation of neuronal markers by neuron‐specific expression of GFP. (A) Flow cytometry plot showing CD45 and various neuronal markers. Papain‐treated P8 telencephalons were used in this study. The percentage of cells that were CD45 negative and recognized by each antibody is shown in red. (B) Representative gating strategy. Green fluorescent protein (GFP) was expressed by the Adeno‐associated virus (AAV) under the Neuron‐specific enolase (NSE) promoter. Virus injection was performed at P21, and a papain‐treated P35 telencephalon was used for flow cytometry. The percentage of cells that were positive for both CD200 and GFP is shown in red. (C) Flow cytometry plots showing signals in unstained and uninjected cells. (D) Flow cytometry plot showing GFP and various neuronal markers. The percentage of cells positive for both GFP and each neuronal marker is shown in red. (E) Comparison of the percentage of GFP‐positive and GFP‐negative cells stained with various neuronal markers (*n* = 4 for each bar). Error bars represent mean ± SEM. The *P*‐values of the Student's *t*‐test are indicated.

We searched for antibodies that stain a wide range of neurons. Because neurons are CD45 negative, as the first step of screening, we used flow cytometry to identify antibodies that reacted with many CD45‐negative cells in a specific manner (Fig. 3A). The results showed that antibodies against microtubule‐associated protein 2a + 2b (MAP2ab), CD200, neural cell adhesion molecule (NCAM), NeuN, α‐synuclein, Synapsin I, and GAD65 specifically stained many CD45‐negative cells compared to the control (Fig. 3A).

Furthermore, we investigated whether these antibodies correctly recognized neurons by flow cytometry. Green fluorescent protein (GFP) was expressed in AAV under the neuron‐specific enolase (NSE) promoter, and neurons were identified using GFP (Fig. 3B). CD31‐positive vascular endothelial cells were excluded because they take up endogenous IgG [13] and react with anti‐mouse IgG, resulting in false positives (Fig. 3B). CD45‐positive cells were also excluded because microglia and macrophages, which are CD45‐positive, also take up endogenous antibodies [14]. The percentages of GFP‐positive and GFP‐negative cells stained with these antibodies were compared (Fig. 3C–E). There was no statistically significant difference between the percentage of GFP‐positive cells stained with anti‐MAP2ab antibody and the percentage of GFP‐negative cells stained with anti‐MAP2ab antibody, while the other three antibodies showed statistically significant differences (Fig. 3E). Therefore, the anti‐CD200, NCAM, and NeuN antibodies used in this study were found to recognize neurons by flow cytometry.

NCAM is a transmembrane protein [15]. Therefore, we examined whether anti‐NCAM antibodies can recognize neurons without cell membrane permeabilization. The results showed that anti‐NCAM antibodies recognized neurons when subjected to cell membrane permeabilization (Fig. S1A,C), but not when cells were not treated with cell membrane permeabilization (Fig. S1B,C).

**Fig. 4:**
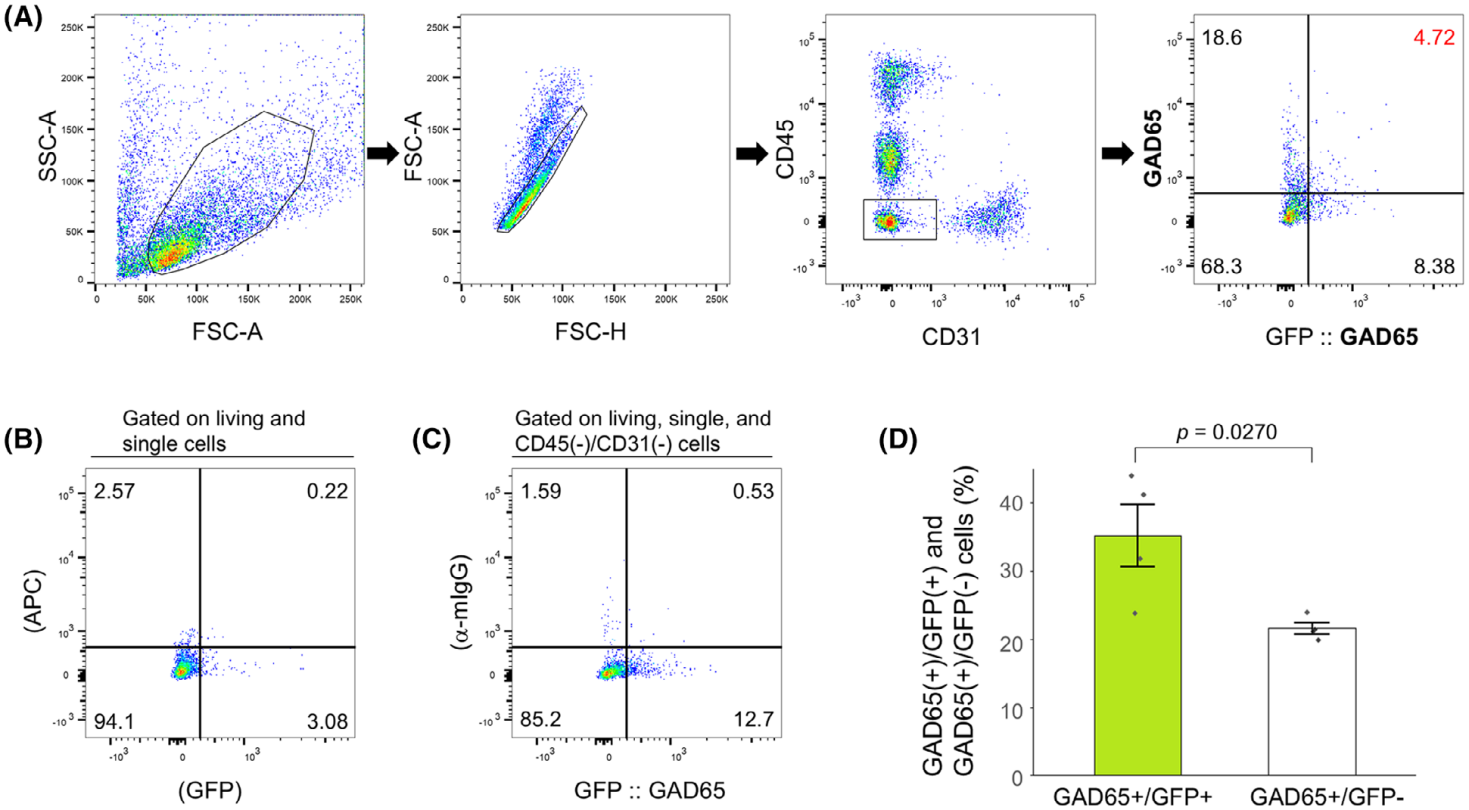
Validation of anti‐GAD65 antibody by AAV injection. (A) Representative gating strategy. GFP was expressed by AAV under the GAD65 promoter. Virus injection was performed at P21, and papain‐treated P35 telencephalon was used in this study. The percentage of cells that were positive for both GFP and anti‐GAD65 antibodies is shown in red. (B) Flow cytometry plot showing signals in unstained and uninjected cells. (C) Flow cytometry plots displaying GFP signals and anti‐mouse IgG secondary antibody. (D) Comparison of the percentage of GFP‐positive and GFP‐negative cells stained with anti‐GAD65 antibody (*n* = 4 for each bar). Error bars represent mean ± SEM. The *P*‐values of the Student's *t*‐test are indicated.

The same strategy was used to evaluate GAD65 antibodies; AAV was used to express GFP under the control of the GAD65 promoter (Fig. 4A–C). The percentages of GFP‐positive and GFP‐negative cells stained with anti‐GAD65 antibody were compared (Fig. 4D). The results showed that the anti‐GAD65 antibody specifically recognized GAD65‐positive neurons by flow cytometry. These results indicate that various neuronal markers must be used appropriately for different purposes (Table 1).

**Table 1:** Comparison of neuronal markers.

Protein	Validity	Cellular localization	Additional information
NCAM	+++	Membrane	Requires cell membrane permeabilization
NeuN	++	Nuclear	The optimization of cell membrane permeabilization parameters is crucial
CD200	+	Membrane	No cell membrane permeabilization required
GAD65	+	Cytosol	The optimization of cell membrane permeabilization parameters is crucial
MAP2ab	−	Cytosol	

### Differences in protease and survival of various brain cells

**Fig. 5:**
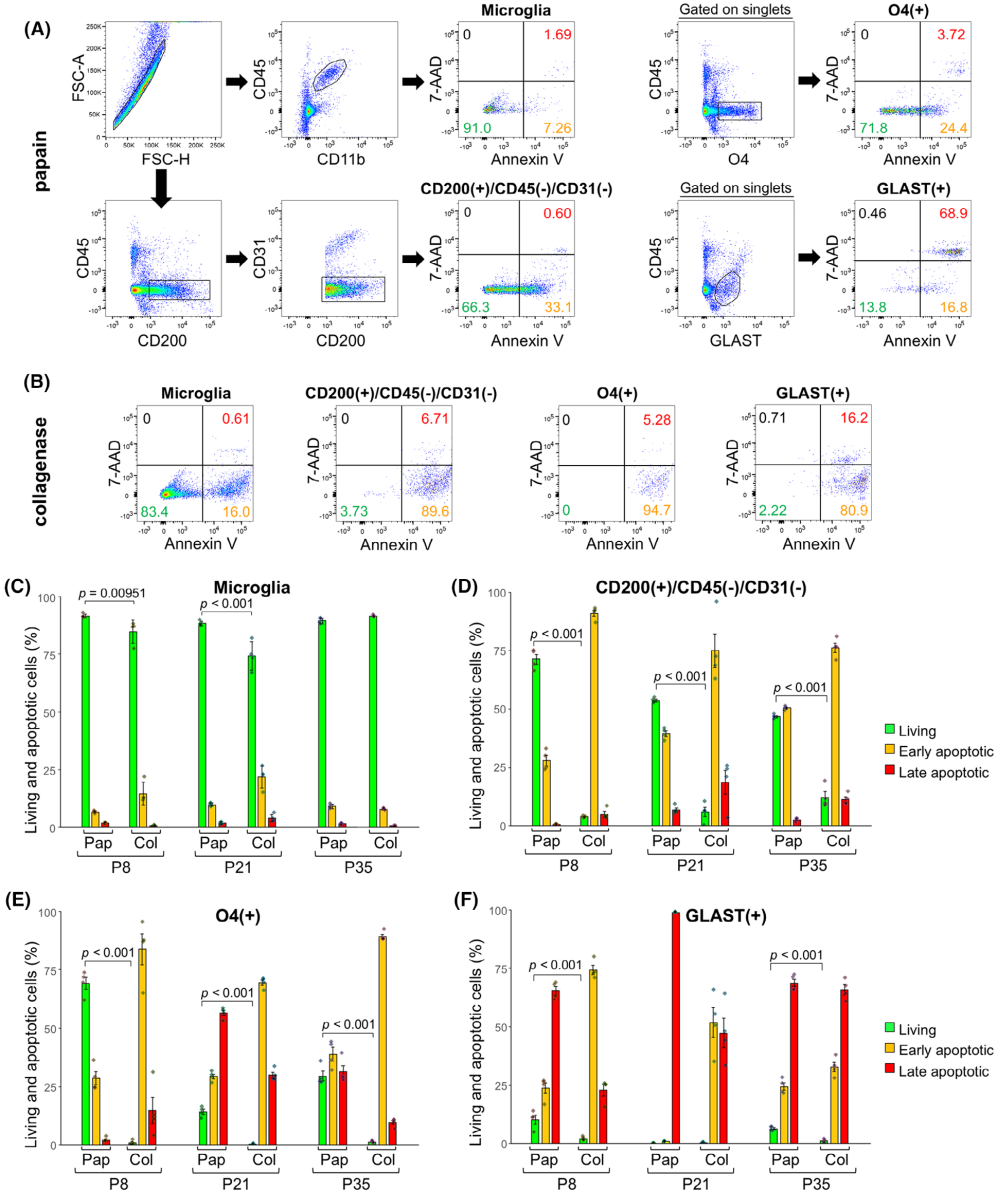
Comparison of apoptotic cells prepared by papain and collagenase treatments. (A) Gating strategy to assess apoptosis in papain‐treated brain cells. The telencephalon of mice at P8 was used for flow cytometry. Annexin V‐positive/7‐Amino‐Actinomycin D (7‐AAD)‐negative cells were in the early stage of apoptosis, whereas double‐positive cells were in the later stage of apoptosis. The percentages of living, early apoptotic, and late apoptotic cells are shown in green, orange, and red, respectively. (B) Apoptosis of collagenase‐treated cells prepared from the P8 telencephalon. (C–F) Apoptosis of microglia (C), CD200‐positive neurons (D), O4‐positive oligodendrocytes (E), and Glutamate aspartate transporter (GLAST)‐positive astrocytes (F) by papain and collagenase treatment. The percentages of living, early apoptotic, and late apoptotic cells are shown in green, orange, and red, respectively (*n* = 4 for each bar). A two‐way analysis of variance revealed a significant effect of protease (*P* < 0.0001) and stage (*P* < 0.0001) in living cells of all cell types. The *P*‐values of Fisher's PLSD *post‐hoc* tests are indicated. Error bars represent mean ± SD.

Many studies have used collagenase for flow cytometry analysis of the brain [4, 11, 16, 17], and papain has also been used [18]. We compared the two methods with respect to cell viability using an apoptosis assay. Annexin V binds to phosphatidylserine, which is exteriorized to the cell membrane after apoptosis [19]. Since Annexin V is used in the apoptosis assay, various brain cells were identified using antibodies raised against CD200, oligodendrocyte marker O4, glutamate aspartate transporter (GLAST), CD11b, and CD45, which recognize cells without cell membrane permeabilization. The FSC‐SSC scatter profile was not used to exclude apoptotic cells (Fig. 5A). Annexin V‐positive/7‐amino‐actinomycin D (7‐AAD)‐negative cells were determined to be in the early stages of apoptosis, whereas double‐positive cells were in the later stages (Fig. 5A,B), as previously reported [20].

For microglia, the percentage of living cells was high regardless of the type of protease or the developmental stage (Fig. 5C). In CD200‐positive neurons, the percentage of living cells was higher after papain treatment than after collagenase treatment (Fig. 5D). O4‐positive oligodendrocytes also showed a higher percentage of living cells in the papain‐treated group than in the collagenase‐treated group, and there were more living cells in the earlier stages (Fig. 5E). Most GLAST‐positive astrocytes were apoptotic, regardless of the type of enzyme (Fig. 5F).

**Fig. 6:**
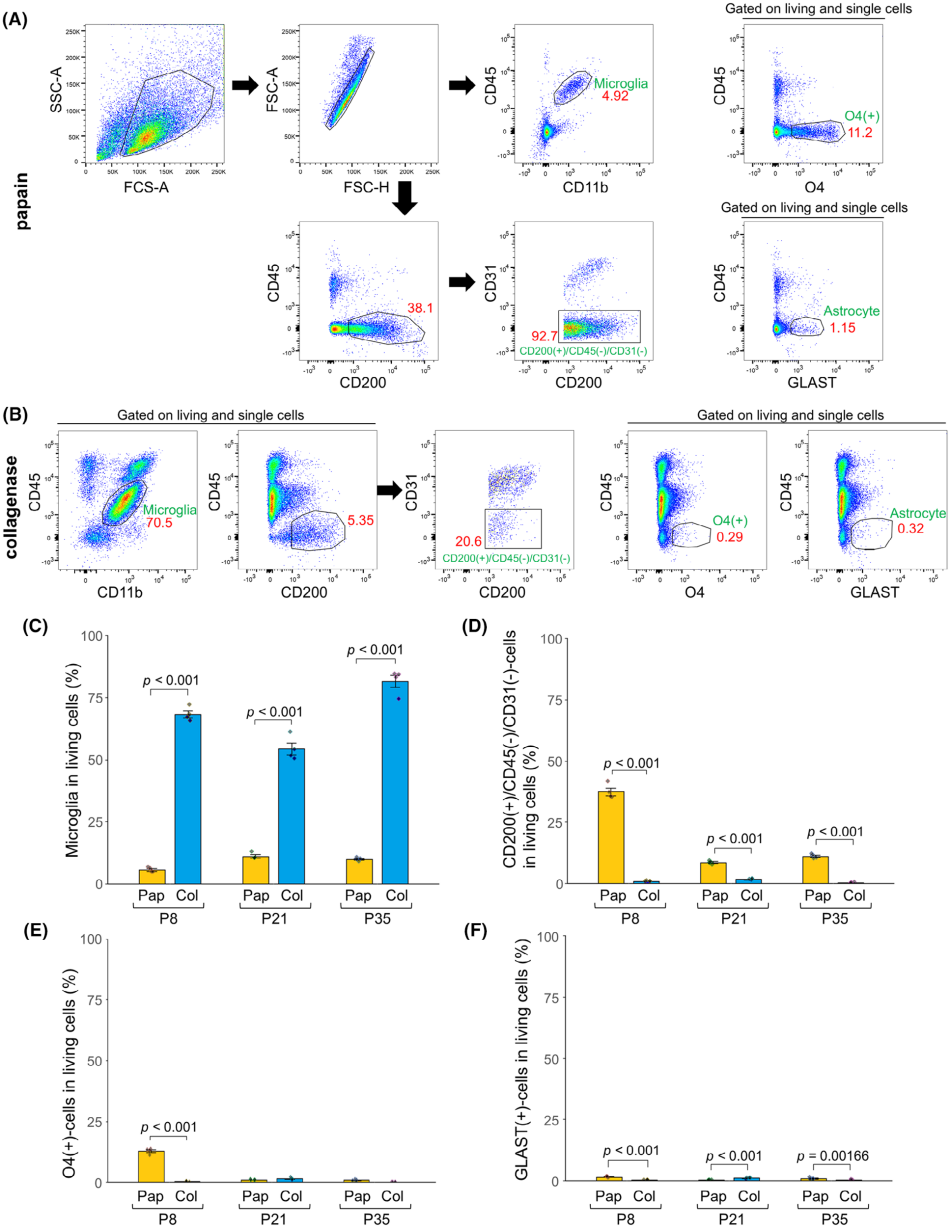
Protease‐dependent differences in recovery rates of various brain cells. (A) Gating strategy for determining the yield of various brain cells after papain treatment. The telencephalon of mice at P8 was used for flow cytometry. (B) Flow cytometry plots showing various types of cells prepared by collagenase treatment. (C) Microglia yields after papain and collagenase treatment. Papain‐ and collagenase‐treated cells are shown in yellow and blue, respectively (*n* = 4 for each bar). Two‐way analysis of variance revealed a significant effect of protease (*P* < 0.0001) and stage (*P* < 0.0001). The *P*‐values of Fisher's PLSD *post‐hoc* tests are indicated. (D) Yield of CD200‐positive neurons after papain and collagenase treatment (*n* = 4 for each bar). Two‐way analysis of variance revealed a significant effect of protease (*P* < 0.0001) and stage (*P* < 0.0001). (E) Yield of O4‐positive oligodendrocytes after papain and collagenase treatment (*n* = 4 for each bar). Two‐way analysis of variance revealed a significant effect of protease (*P* < 0.0001) and stage (*P* < 0.0001). (F) Yield of GLAST‐positive astrocytes after papain and collagenase treatment (*n* = 4 for each bar). Two‐way analysis of variance revealed a significant effect of protease (*P* = 0.00211) and stage (*P* = 0.0470). Error bars represent mean ± SEM.

Next, we examined the effect of the protease type on the yield of various cell types. Debris and dead cells were excluded using the FSC‐SSC scatter profiles (Fig. 6A). Following collagenase treatment, more than half of the cells collected in all developmental stages were microglia (Fig. 6B,C). CD200‐positive neurons were poorly isolated by collagenase digestion, while papain treatment resulted in an improved yield (Fig. 6D). A high percentage of O4‐positive oligodendrocytes was collected only with papain treatment at P8 (Fig. 6E). Neither collagenase nor papain treatment was effective in collecting astrocytes (Fig. 6F).

**Fig. 7:**
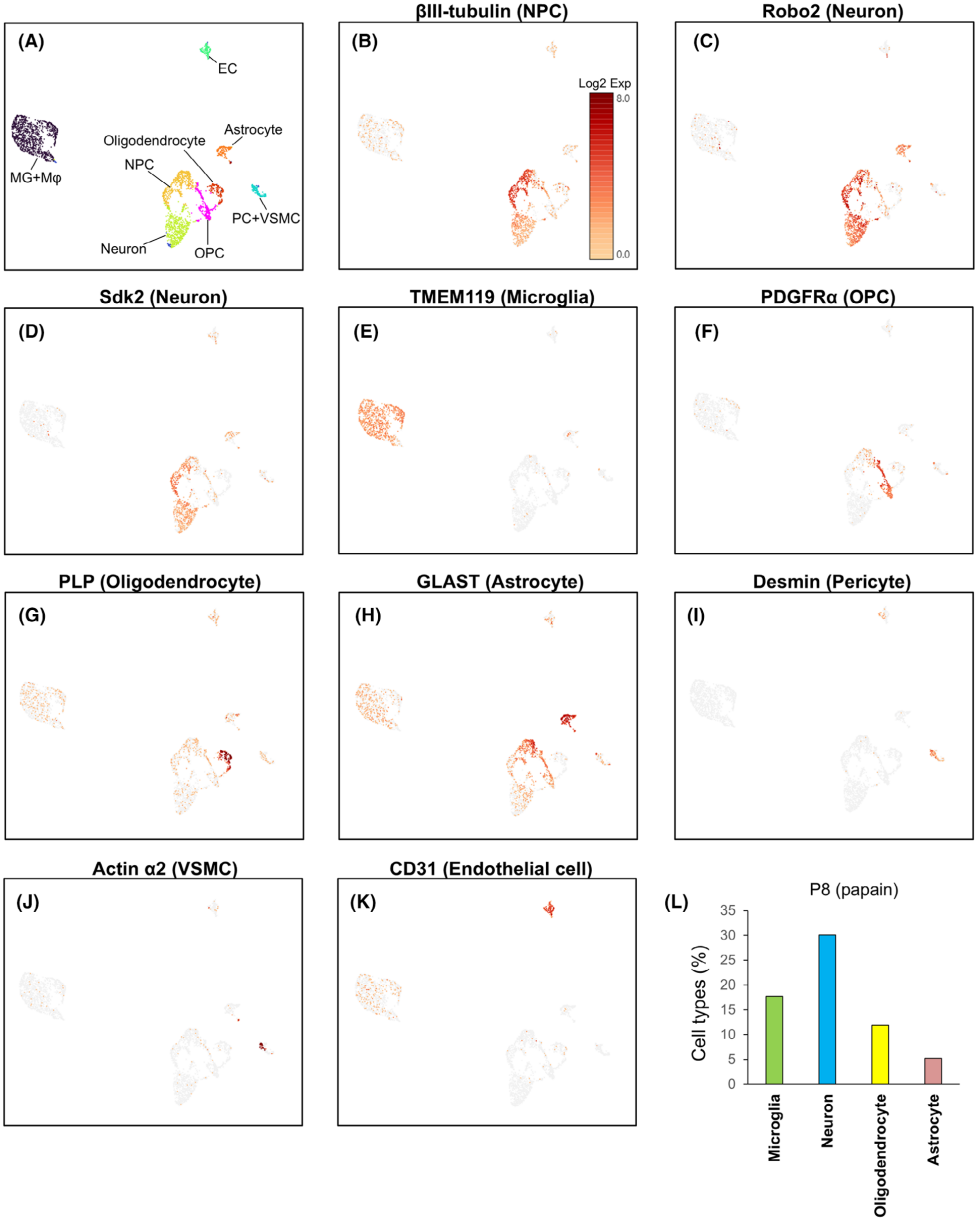
Identification of the cell types prepared by papain treatment using single‐cell RNA sequencing (scRNA‐Seq). (A) UMAP visualization of clustering results for brain cells. P8 telencephalon cells were treated with papain, and cell types were determined using gene expression profiles. (B–K) The expression levels of genes specific to each cell type are shown in the heat map. Neural precursor cells (NPC) (B), neurons (C, D), microglia (E), oligodendrocyte precursor cells (OPC) (F), oligodendrocytes (G), astrocytes (H), pericytes (I), vascular smooth muscle cells (VSMC) (J), and endothelial cells (K) are shown. (L) Percentage of cell types prepared by papain treatment of P8 telencephalon. The data of the scRNA‐Seq experiment is from a single experiment.

We used single‐cell RNA sequencing (scRNA‐seq) to verify whether the percentages of the various cells identified using antibodies were reasonable. Brain cell types that were prepared by papain treatment at P8 were identified using gene expression profiling (Fig. 7A). The results showed that papain treatment was effective in collecting pericytes (Fig. 7I), vascular smooth muscle cells (VSMCs) (Fig. 7J), and endothelial cells (Fig. 7K), in addition to neurons (Fig. 7B–D), microglia (Fig. 7E), oligodendrocytes (Fig. 7F,G), and astrocytes (Fig. 7H). The percentages of microglia, neurons, and oligodendrocytes (Fig. 7L) were generally similar to those determined using flow cytometry at P8 (Fig. 6C–F). By contrast, the percentage of astrocytes determined by scRNA‐Seq was 5.22% (Fig. 7L), whereas that determined by flow cytometry was only 1.58% (Fig. 6F). In the experiment performed in Fig. 5, the percentage of astrocytes was 3.05% when considering both early and late apoptotic cells. Therefore, we speculate that scRNA‐Seq identifies apoptotic astrocytes, but the results of flow cytometry exclude many astrocytes when the gate was used to select living cells.

Because of this tendency of astrocytes to undergo apoptosis during cell dissociation, we attempted to enhance cell survival rates of the cells by collecting them under gentler conditions. Brain cells were collected after 30 min of accutase treatment on ice, and the number that had undergone apoptosis was analyzed using flow cytometry (Fig. S2). Live microglial cells were evident (Fig. S2B), but few live neurons (Fig. S2C), oligodendrocytes (Fig. S2D), and astrocytes were observed (Fig. S2E). With regard to the enzyme treatment effect on astrocyte apoptosis, accutase was more damaging than papain and comparable to collagenase (Fig. 5F).

Next, we examined the effect of accutase treatment on the yield of various cell types (Fig. S3). Debris and dead cells were excluded using the FSC‐SSC scatter profiles (Fig. S3A). The results showed that most of the collected cells were microglia (Fig. S3A,B). Therefore, accutase is not suitable for collecting astrocytes (Fig. S3E). Table 2 displays the effects of papain, collagenase, and accutase treatments on the preparation of different cell types.

**Table 2:** Evaluation of cell type and enzyme combinations.

Cell type	Papain	Collagenase	Accutase	Additional information
Neurons	++	−	−	Anti‐NCAM antibody is recommended when membrane permeabilization is possible
Microglia	+	+++	++	The percentage of microglia in all collected cells is significantly lower in the papain treatment
Oligodendrocytes	+	−	−	The yield of O4‐positive cells is significantly lower in later developmental stages
Astrocytes	−	−	−	

### Differences in cells recognized by various neuronal markers in the developmental stage

**Fig. 8:**
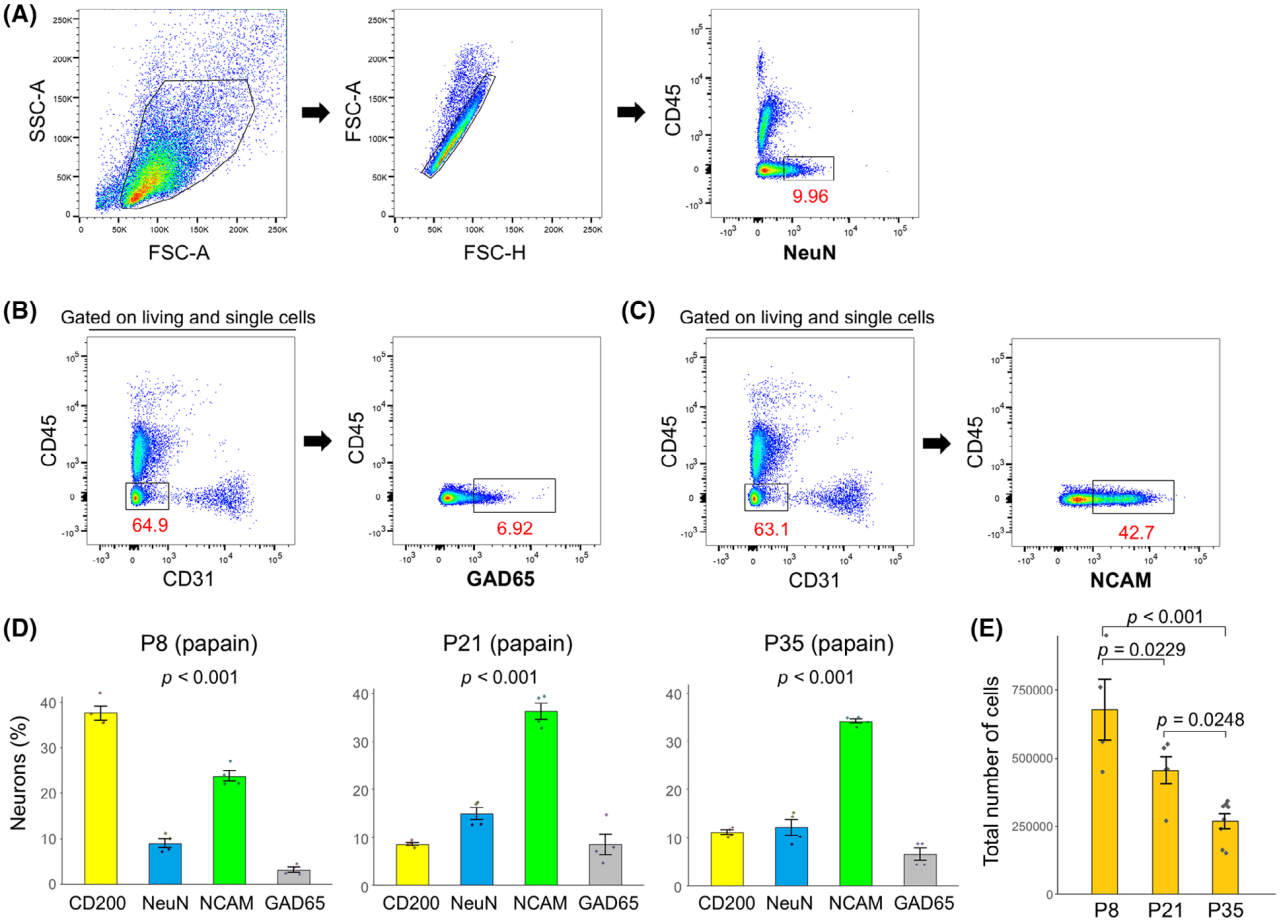
Differences in the number of cells recognized by various neuronal markers. (A–C) Gating strategy used to identify NeuN‐positive (A), GAD65‐positive (B), and Neural cell adhesion molecule (NCAM)‐positive (C) neurons. In this study, the papain‐treated P8 telencephalon was used. The gating strategy for CD200‐positive neurons was the same as that shown in Fig. 6A. (D) Percentage of cells labeled with various neuronal markers at P8, P21, and P35 (*n* = 4 for each bar). Error bars represent mean ± SEM. One‐way analysis of variance showed a significant difference (*P* < 0.0001). (E) The number of total cells collected after papain treatment of the telencephalon at P8 (*n* = 4), P21 (*n* = 5), and P35 (*n* = 8). Error bars represent mean ± SEM. One‐way analysis of variance showed a significant difference (*P* < 0.0001). The *P*‐values of Fisher's PLSD *post‐hoc* tests are indicated.

We also examined differences in the percentage of cells recognized by various neuronal markers when treated with papain (Fig. 8A–C). A higher percentage of CD200‐positive neurons was observed at P8, while a higher percentage of NCAM‐positive neurons was observed at P21 and P35 (Fig. 8D). In addition, the results showed that the total brain cell yield tended to be lower in later developmental stages (Fig. 8E).

**Fig. 9:**
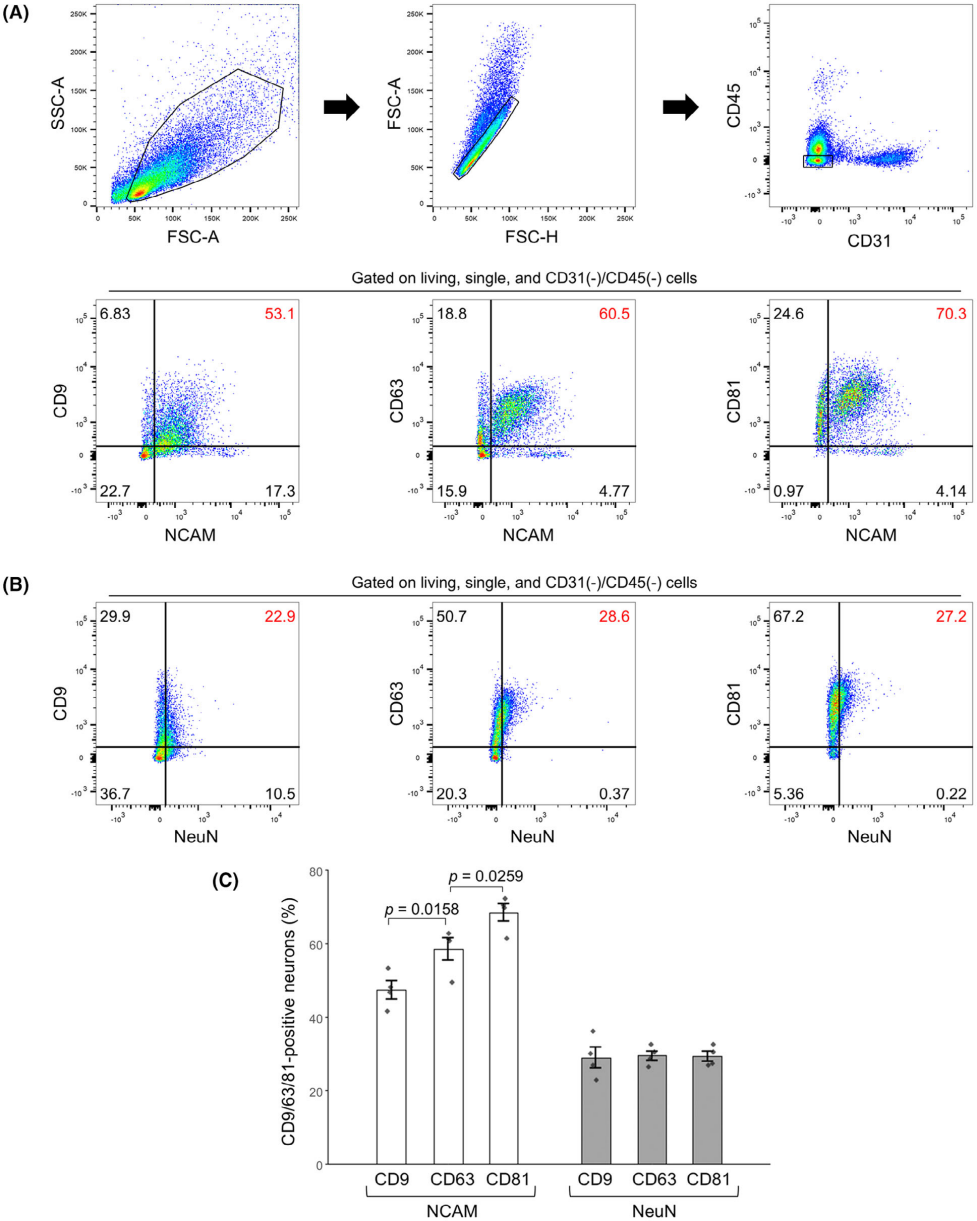
The number of various exosome types in NCAM‐ and NeuN‐positive neurons. (A) Gating strategy to analyze the types of exosomes contained in NCAM‐positive cells. In this study, the papain‐treated P8 telencephalon was used. CD9, CD63, and CD81 were used as exosome markers. The percentage of cells that were positive for both exosome markers and NCAM is shown in red. (B) Types of exosomes contained in NeuN‐positive cells. The percentage of cells that were positive for both exosome markers and NeuN is shown in red. (C) Percentage of NCAM‐positive (white bar) and NeuN‐positive cells (gray bars) that were positive for various exosome markers (*n* = 4 for each bar). Error bars represent mean ± SEM. The two‐way analysis of variance revealed a significant effect of neural markers (*P* < 0.0001) and exosome markers (*P* < 0.0001). The *P*‐values of Fisher's PLSD *post‐hoc* tests are indicated.

We examined whether differences in these neuronal markers affected the results. The types of exosomes present in NCAM‐positive neurons (Fig. 9A) and NeuN‐positive neurons (Fig. 9B) were compared by flow cytometry with endosome‐specific tetraspanins (CD9, CD63, and CD81) [21]. Interestingly, there was a significant difference in the number of NCAM‐positive neurons that were positive for CD9, CD63, and CD81, but not in the number of NeuN‐positive neurons (Fig. 9C).

## Discussion

Neurons and oligodendrocytes are known to have a greater susceptibility to damage compared to microglia [22]. Therefore, an important step in flow cytometry is the preparation of a viable cell suspension that limits cell damage and/or death. Various strategies have been used to prepare neurons for flow cytometry, including collagenase [17], papain [18], accutase [23, 24], Dounce homogenizers [25, 26], and commercially available kits [27]. On the other hand, a Percoll gradient has been used to remove cell debris and myelin from these dissociated adult CNS tissue preparations [28, 29]. Others have used a single 30–37% Percoll step to isolate microglia and leukocytes [4, 8, 9, 10, 11, 12]. We optimized a brain preparation protocol using papain + DNase I digestion, followed by centrifugation with low concentrations of Percoll, which permitted maximal cell yield and viability and optimal detection of both neuronal and non‐neuronal cells in mice.

In flow cytometry, autofluorescence is a troublesome phenomenon that masks the correct fluorescence signals. Lipofuscin is the largest source of autofluorescence in brain tissue. Lipofuscin is a mixture of highly oxidized lipids, misfolded proteins, and metals that accumulate with age within the lysosomal compartments [30, 31, 32]. In microglia, lipofuscin aggregation can be induced by incomplete myelin digestion and disruption of the lysosomal pathway, which implicates phagocytosis of cellular material as a key mechanism leading to increased lipofuscin burden [33]. In this study, autofluorescence was compared among the four regions of the mouse infant brain. In the later stages of brain development, lipofuscin accumulates in cell types other than microglia, and the degree of accumulation in gray and white matter also changes [34]. Therefore, when analyzing a more mature brain, careful consideration must be given to not only differences in brain regions but also to whether gray or white matter is to be used and what cell types are to be analyzed.

The number of flow cytometric markers available for brain cell analysis on the market is limited, in part due to the lack of previous demand. NeuN is the only neuronal marker used for flow cytometry [17, 25, 26]. Anti‐NCAM, CD200, and GAD65 antibodies used in this study were also useful for identifying neurons by flow cytometry. Our results indicated that differences between neuronal markers may lead to different results in some assays (Fig. 9). For various flow cytometry assays, it is necessary to validate the results using multiple neuronal markers.

In this study, we aimed to determine the optimal conditions for flow cytometry analysis of mouse brains. These conditions need to be optimized for other species. In particular, fixed samples are used when analyzing human brains. A previous study showed no difference in flow cytometry results between fresh and frozen tissues [35]. However, in that study, the percentage of living cells in fresh tissue was as low as 1.9% [35], whereas that in our analysis was approximately 70% (Fig. 6A, leftmost panel). Therefore, whether the experimental method yields different results between fresh and fixed samples needs to be clarified.

The flow cytometric method has become increasingly widespread recently but has been hampered by challenges when applied to neuroscience. We found that the type of protease, brain region, and developmental stage used, as well as the type of neuronal marker used influenced the results. Considering these factors, flow cytometry can be applied to the brain to quantify various activities in large amounts in a short time.

## Materials and methods

### Mice

Mice were housed two to three per cage in a room with a 12‐h light/dark cycle (lights on at 7:00 am), with access to food and water *ad libitum*. The animals were cared for and treated according to the “Japanese Act on the Welfare and Management of Animals,” the “Guidelines for the Proper Conduct of Animal Experiments” issued by the Science Council of Japan, and the National Research Council's “Guide for the Care and Use of Laboratory Animals.” All experimental protocols were reviewed and approved by the Animal Care and Experimentation Committee of Gunma University.

### Flow cytometry

Previous methods to treat the brain with collagenase were modified [4, 14]. The ages of the mice used in each experiment are described in the figure legend. Unless otherwise noted, male and female mice were used. The neocortices, hippocampi, and corpus callosum of C57BL/6J mice (Nippon SLC, Shizuoka, Japan) were dissected, minced, and incubated with 500 U of type II collagenase (4176, Worthington Biochemical Corp, Freehold, NJ, USA) and 250 U of DNase (D4527; Millipore‐Sigma, St. Louis, MO, USA) for 30 min at 37 °C in 10 mL of Hanks' balanced salt solution (HBSS). Cells were triturated by repeated passage through 1 mL and 200 μL plastic micropipette tips every 5 min during incubation. After incubation, cells were filtered through a 70 μm cell strainer (3‐6649‐02; ASONE, Osaka, Japan) and pelleted by centrifugation (400 ***g*** for 5 min at 18 °C).

A previously reported method for treating the brain with papain was used with some modifications [14]. The neocortices, hippocampi, and corpus callosum of C57BL/6j mice were dissected, minced, and digested with 45 U papain (PAPL, Worthington), 0.1 kU·mL^−1^ DNase I, 0.02% DL‐cysteine, 0.02% bovine serum albumin (BSA), and 0.5% glucose in phosphate‐buffered saline (PBS) at 200 rpm for 10 min at 37 °C. Fetal bovine serum (FBS) was added at a final concentration of 20% to inhibit papain activity. After washing twice with HBSS, cells were triturated by repeated passage through 1 mL and 200 μL plastic micropipette tips on ice, filtered through a 70 μm cell strainer (3‐6649‐02; ASONE), and pelleted in a centrifuge (400 ***g***, 5 min, 18 °C).

A previously reported method for treating the brain with accutase was used, with some modifications [23, 24]. The neocortices, hippocampi, and corpus callosum of C57BL/6j mice were dissected, minced, and digested with 2 mL of accutase (SCR005; Millipore‐Sigma) for 30 min on ice with gentle shaking. The cells were triturated by repeated passage through 1 mL and 200 μL plastic micropipette tips on ice, filtered through a 70 μm cell strainer (3‐6649‐02; ASONE), and pelleted in a centrifuge (400 ***g***, 5 min, 18 °C).

Nine parts of Percoll (17 089 102, Cytiva, Marlborough, MA, USA) and one part of 10 × PBS were mixed to prepare a 100% stock isotonic Percoll (SIP) solution. When the concentration of 100% SIP was reduced, the solution was diluted with PBS. Cell pellets were resuspended in 24% SIP, overlaid with PBS, and centrifuged (400 ***g*** for 25 min at 18 °C). After centrifugation, the supernatant was discarded, and erythrocytes were lysed using RBC lysis buffer (420 310; BioLegend). Five times the volume of PBS was added to the RBC lysis buffer and centrifuged at 400 ***g*** for 5 min. The precipitated cells were suspended in HBSS and counted. The 0.1% BSA–PBS containing 5 μg·mL^−1^ of rat anti‐CD16/32 antibody (C247; Leinco Technology, St. Louis, MO, USA) was used for blocking. Unfixed cells were stained with cell surface antigens prior to fixation. Details of antibodies used in this study can be found in Table 3. Intracellular antigens were stained using Fixation Buffer (420 801; BioLegend, San Diego, CA, USA) and Intracellular Staining Permeabilization Wash Buffer (421 002; BioLegend), according to the manufacturer's instructions. PE streptavidin (425 203; BioLegend) or FITC streptavidin (405 201; BioLegend) was used to label biotinylated antibodies with fluorescence according to the manufacturer's instructions. Goat polyclonal anti‐mouse IgG (APC) (405 308; BioLegend) or goat polyclonal anti‐mouse IgG (Alexa Fluor^®^ 488) (A31620; Thermo Fisher Scientific Waltham, MA, USA) was used as a secondary antibody for mouse primary antibody without fluorescent labeling. Annexin V/7‐AAD staining was performed using the PE Annexin V Apoptosis Detection Kit with 7‐AAD (640 934; BioLegend) according to the manufacturer's instructions.

**Table 3:** Key resources table.

Reagent type	Designation	Source or reference	Identifiers	Additional information
Antibody	Anti‐CD45 (PerCP/Cy5.5) (rat monoclonal)	BioLegend	Cat# 101217	FC (1:200)
Antibody	Anti‐CD45 (APC/Cy7) (rat monoclonal)	BioLegend	Cat# 103115	FC (1:200)
Antibody	Anti‐mouse IgG H&L (Alexa Fluor^®^ 488) (goat polyclonal)	Thermo Ficher Scientific	Cat# A31620	FC (1:400)
Antibody	Anti‐mouse IgG (APC) (goat polyclonal)	BioLegend	Cat# 405308	FC (1:100)
Antibody	Anti‐MAP2 (2a + 2b) (mouse monoclonal)	Millipore‐Sigma	Cat# M1406	FC (10 ng·μL^−1^)
Antibody	Anti‐CD200 (PE/Cy7) (rat monoclonal)	BioLegend	Cat# 123817	FC (1:200)
Antibody	Anti‐CD200 (APC) (rat monoclonal)	BioLegend	Cat# 123809	FC (1:200)
Antibody	Anti‐NCAM (mouse monoclonal)	Millipore‐Sigma	Cat# C9672	FC (10 ng·μL^−1^)
Antibody	Anti‐NeuN (biotin) (mouse monoclonal)	Millipore‐Sigma	Cat# MAB377B	FC (5 ng·μL^−1^)
Antibody	Anti‐α‐Synuclein (mouse monoclonal)	BD Biosciences	Cat# 610786	FC (2.5 ng·μL^−1^)
Antibody	Anti‐Synapsin I (mouse monoclonal)	Synaptic Systems	Cat# 106011	FC (10 ng·μL^−1^)
Antibody	Anti‐GAD65 (mouse monoclonal)	Developmental Studies Hybridoma Bank	Cat# GAD‐6	FC (2 ng·μL^−1^)
Antibody	Anti‐synaptophysin (mouse monoclonal)	Millipore‐Sigma	Cat# S5768	FC (10 ng·μL^−1^)
Antibody	Anti‐doublecortin (mouse monoclonal)	BD Biosciences	Cat# 611706	FC (2.5 ng·μL^−1^)
Antibody	Anti‐tyrosine hydrolase (mouse monoclonal)	Millipore‐Sigma	Cat# MAB318	FC (1:100)
Antibody	Anti‐GAD67 (mouse monoclonal)	Millipore‐Sigma	Cat# MAB5406	FC (10 ng·μL^−1^)
Antibody	Anti‐calbindin (mouse monoclonal)	Millipore‐Sigma	Cat# C9848	FC (10 ng·μL^−1^)
Antibody	Anti‐parvalbumin (mouse monoclonal)	Millipore‐Sigma	Cat# P3088	FC (10 ng·μL^−1^)
Antibody	Anti‐CD31 (PE/Cy7) (rat monoclonal)	BioLegend	Cat# 102523	FC (1:200)
Antibody	Anti‐CD31 (APC) (rat monoclonal)	BioLegend	Cat# 102509	FC (1:200)
Antibody	Anti‐CD11b (Alexa Fluor^®^ 488) (rat monoclonal)	BioLegend	Cat# 101217	FC (1:200)
Antibody	Anti‐O4 (APC) (mouse monoclonal)	Miltenyi Biotec	Cat# 130–119‐155	FC (1:400)
Antibody	Anti‐GLAST (APC) (mouse monoclonal)	Miltenyi Biotec	Cat# 130–123‐641	FC (1:400)
Antibody	Anti‐CD9 (PE) (rat monoclonal)	BioLegend	Cat# 124805	FC (1:200)
Antibody	Anti‐CD63 (PE) (rat monoclonal)	BioLegend	Cat# 143903	FC (1:200)
Antibody	Anti‐CD81 (PE) (hamster monoclonal)	BioLegend	Cat# 104905	FC (1:200)

Staining was measured using a BD FACSCanto II flow cytometer (BD Biosciences, San Jose, CA, USA) and analyzed using FlowJo software (BD Biosciences). Unless otherwise noted, initial gating was performed on live and singlet cells.

### Adeno‐associated virus (AAV) vector

The recombinant single‐stranded AAV‐PHP.eB vector [36] was produced using an ultracentrifugation method, as previously reported [37]. Briefly, three plasmids were used: an expression plasmid (pAAV/NSE‐GFP [38] or pAAV/mGAD65‐GFP [39]), pHelper (Agilent Technologies, Santa Clara, CA, USA), and the Rep/Cap plasmid pAAV‐PHP.eB were co‐transfected with polyethylenimine (24765–1, Polysciences, Inc., Warrington, PA, USA) into HEK293T cells (HCL4517, Thermo Fisher Scientific) cultured in Dulbecco's modified Eagle's medium (DMEM) (D5796; Millipore‐Sigma) supplemented with 8% FBS. The viral particles were harvested from the culture medium 6 days after transfection and precipitated with 8% polyethylene glycol (P5413; Millipore‐Sigma) and 500 mm sodium chloride. Precipitated AAV particles were resuspended in PBS and purified by iodixanol linear density gradient centrifugation using an ultracentrifuge CP80WX (Himac, Tokyo, Japan). The viral solution was further concentrated and formulated in PBS using Vivaspin (VS15T42; Sartorius, Göttingen, Germany). The genomic titers of the purified AAV vectors were determined by quantitative real‐time PCR with primers 5′‐CTGTTGGGCACTGACAATTC‐3′ and 5′‐GAAGGGACGTAGCAGAAGGA‐3′ targeting the WPRE sequence.

For intravenous injection of AAV vectors, C57BL/6J mice were anesthetized on P21 by intraperitoneal injection of ketamine and xylazine. The 100 μL of AAV vector solution (1.0 × 10^13^ vg·mL^−1^) was injected into the retro‐orbital sinus of the mouse using a 0.5 mL syringe with a 30‐gauge needle (08277; Nipro, Osaka, Japan).

### Immunohistochemistry

Immunohistochemistry was performed as previously described [14, 40]. Briefly, P8 C57BL/6J male mice (Nippon SLC) were deeply anesthetized with a combination of midazolam, medetomidine, and butorphanol tartrate and transcardially perfused with PBS, followed by Zamboni's fixative (2% paraformaldehyde in 0.1 m phosphate buffer, pH 7.4, containing 0.2% picric acid). The tissues were dissected, post‐fixed in Zamboni's fixative at 4 °C for 5 h, and cryoprotected by immersion in 15% sucrose in PBS overnight at 4 °C. After embedding in the Tissue‐Tek OCT compound (Sakura Finetek, Tokyo, Japan), tissues were frozen and sectioned to a thickness of 15 μm using a cryostat (CM1950, Leica Microsystems, Frankfurt, Germany) at −18 °C. Sections were air‐dried for 1 h and rinsed three times with PBS. Unstained sections were mounted in DAPI Fluoromount‐G mounting medium (0100‐20; SouthernBiotech, Birmingham, AL, USA) and observed under a fluorescence microscope (BX51; Olympus, Tokyo, Japan) equipped with a CCD camera (VB‐7000; Keyence, Osaka, Japan).

### Single‐cell RNA sequencing (scRNA‐seq)

Briefly, the neocortices, hippocampi, and corpus callosum of P8 C57BL/6J male mice (Nippon SLC, Shizuoka, Japan) were dissected, and cells were collected by the method of collecting cells by papain for flow cytometry and finally filtered with a 30 μm cell strainer (130–098‐458, Miltenyi Biotec, Bergisch Gladbach, Germany) and resuspended in PBS with 0.1% BSA, which were subsequently captured in droplets at a targeted cell recovery of <4000 cells. Libraries were prepared using the 10× Single Cell Chromium Next GEM Single‐Cell 3′ GEM Library & Gel Bead Kit v3.1 (1 000 128; 10× Genomics, Pleasanton, CA, USA) according to the manufacturer's protocol. After reverse transcription and cell barcoding in droplets, the emulsions were broken and the cDNA was amplified. To construct gene expression libraries, the amplified cDNA was fragmented, end‐repaired, PCR‐amplified with sample indexing primers, and purified using SPRIselect beads (B23317; BECKMAN COULTER, Brea, CA, USA). Libraries were converted and sequenced on a DNBSEQ‐G400 (MGI, Guangdong, China) at a sequencing depth of 95 460 read pairs per cell and analyzed using 10× CellRanger ver. 7.1.0 (10× Genomics). Using the Loupe Browser ver. 8.0.0 (10× Genomics), cells with fewer than 500 detected genes were filtered as dying cells or empty droplets. To visualize the datasets, we used the uniform manifold approximation and projection (UMAP). Each cluster was annotated with reference to a previously reported cell‐type‐specific marker gene [41].

### Statistical analyses

Statistical analyses were performed using Excel (Statcel 3, Social Survey Research Information, Tokyo, Japan). All data were checked for compliance with the statistical assumptions of each test, including normal distribution and equal variance between the groups. Differences between groups were analyzed using Student's *t*‐test, one‐way analysis of variance (ANOVA), and two‐way repeated measures ANOVA according to each experimental design. Statistical significance was set at *P* < 0.05. No statistical methods were used to predetermine sample sizes; however, our sample sizes were similar to those reported in previous studies. Further details of the statistical analyses are provided in the figure legends.

## Conflict of interest

The authors declare no conflict of interest.

## Author contributions

MS and TS designed the experiments. MS, AK, and TS performed the experiments. MS, AK, AT, TK, TI, HH, and TS analyzed the data. MS, AK, and TS wrote the manuscript. MS, AK, TK, HH, and TS contributed to the discussion.

## Peer review

The peer review history for this article is available at https://www.webofscience.com/api/gateway/wos/peer‐review/10.1111/febs.70063.

## Supporting information


**Fig. S1.** Cell membrane permeabilization and anti‐NCAM antibody reactivity.
**Fig. S2.** Apoptotic cells prepared by accutase treatment.
**Fig. S3.** Recovery rates of various brain cells after accutase treatment.

## Data Availability

The RNA‐Seq data presented in this manuscript has been deposited in NCBI's Gene Expression Omnibus and is accessible through GEO Series accession number GSE278315.
