# Supplementary material for: Critical factors for flow cytometry analysis of the brain
Source: FEBS J. 2025 Mar 14;292(13):3412–29. doi: 10.1111/febs.70063 (PMC12220843; doi:10.1111/febs.70063)
Supplement: Supplementary file 1 — Fig. S1. Cell membrane permeabilization and anti‐NCAM antibody reactivity. Fig. S2. Apoptotic cells prepared by accutase treatment. Fig. S3. Recovery rates of various brain cells after accutase treatment. [file FEBS-292-3412-s001.pdf]

Supporting Information for

Critical factors for flow cytometry analysis of the brain

Mizuki Sadakata[1], Ayumu Konno[2, 3], Akinori Takase[4], Tetsuhiro Kasamatsu[5], Takatoshi Iijima[6], Hirokazu Hirai[2, 3], Tetsushi Sadakata[1, *]

[1]Education and Research Support Center, Gunma University Graduate School of Medicine, Maebashi, Gunma 371-8511, Japan

[2]Department of Neurophysiology and Neural Repair, Gunma University Graduate School of Medicine, Maebashi, Gunma 371-8511, Japan

[3]Viral Vector Core, Gunma University Initiative for Advanced Research (GIAR), Maebashi, Gunma 371-8511, Japan

[4]Department of Life Science Support, Research Innovation Center, University Hospitals Sector, Tokai University, Isehara, Kanagawa 259-1193, Japan

[5]Department of Medical Technology and Clinical engineering, Gunma University of Health and Welfare, Maebashi, Gunma 371-0823, Japan

[6]Department of Molecular Life Science, Division of Basic Medical Science and Molecular Medicine, School of Medicine, Tokai University, Isehara, Kanagawa 259-1193, Japan

[*]Corresponding author. Tel: +81 27 220 8299; E-mail: sadakata-1024@gunma-u.ac.jp

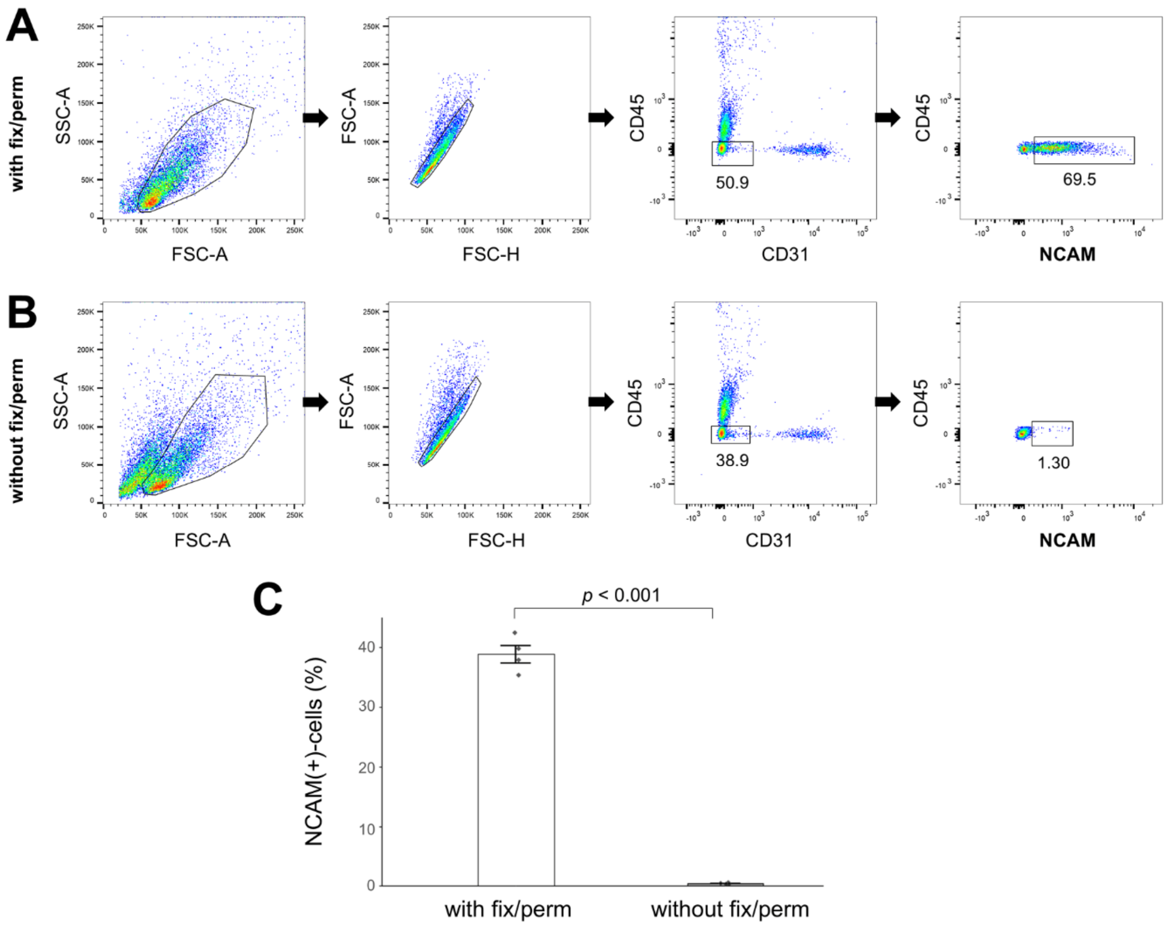

Fig. S1. Cell membrane permeabilization and anti-NCAM antibody reactivity.

(A) Representative gating strategy. In this study, the papain-treated P35 telencephalon was used. Cells were fixed, permeabilized, and recognized using an anti-NCAM antibody. **(B)** Cells were not fixed or permeabilized and recognized by anti-NCAM antibodies. **(C)** The percentage of CD45(−)/CD31(−) cells recognized by the anti-NCAM antibody ($n = 4$ for each bar). Error bars represent mean ± SEM. The p-values of the Student's t-test are indicated.

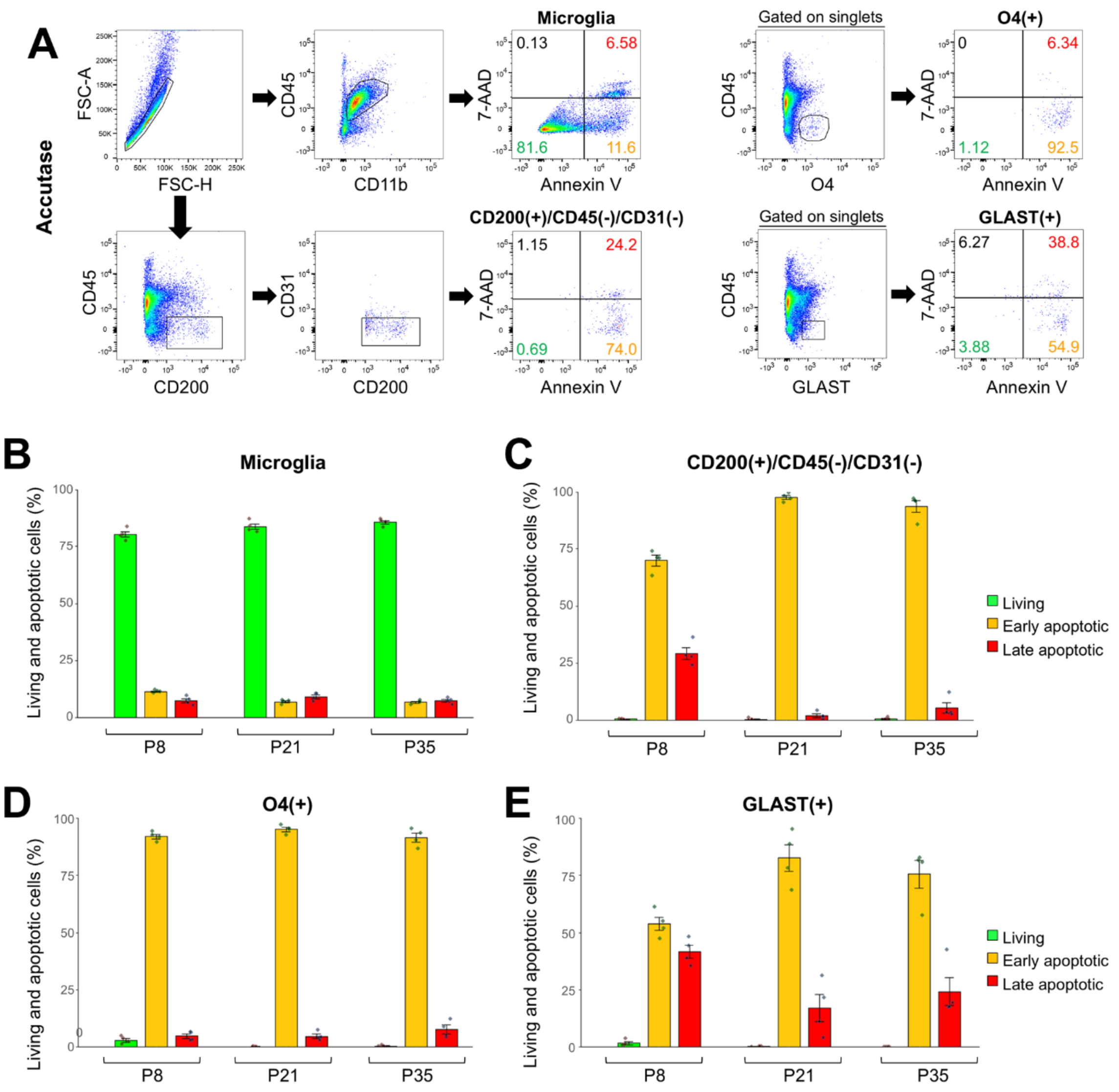

Fig. S2. Apoptotic cells prepared by accutase treatment.

(**A**) Gating strategy for assessing apoptosis in accutase-treated brain cells. The telencephalon of mice at P8 was used for flow cytometry. Annexin V-positive/7-AAD-negative cells were in the early stage of apoptosis, whereas double-positive cells were in the later stage. The percentages of living, early apoptotic, and late apoptotic cells are shown in green, orange, and red, respectively. (**B-E**) Percentages of living and apoptotic microglia (**B**), CD200-positive neurons (**C**), O4-positive oligodendrocytes (**D**), and GLAST-positive astrocytes (**E**) after accutase treatment. The percentages of living, early apoptotic, and late apoptotic cells are shown in green, orange, and red, respectively (n = 4 for each bar). Error bars represent the mean ± SD.

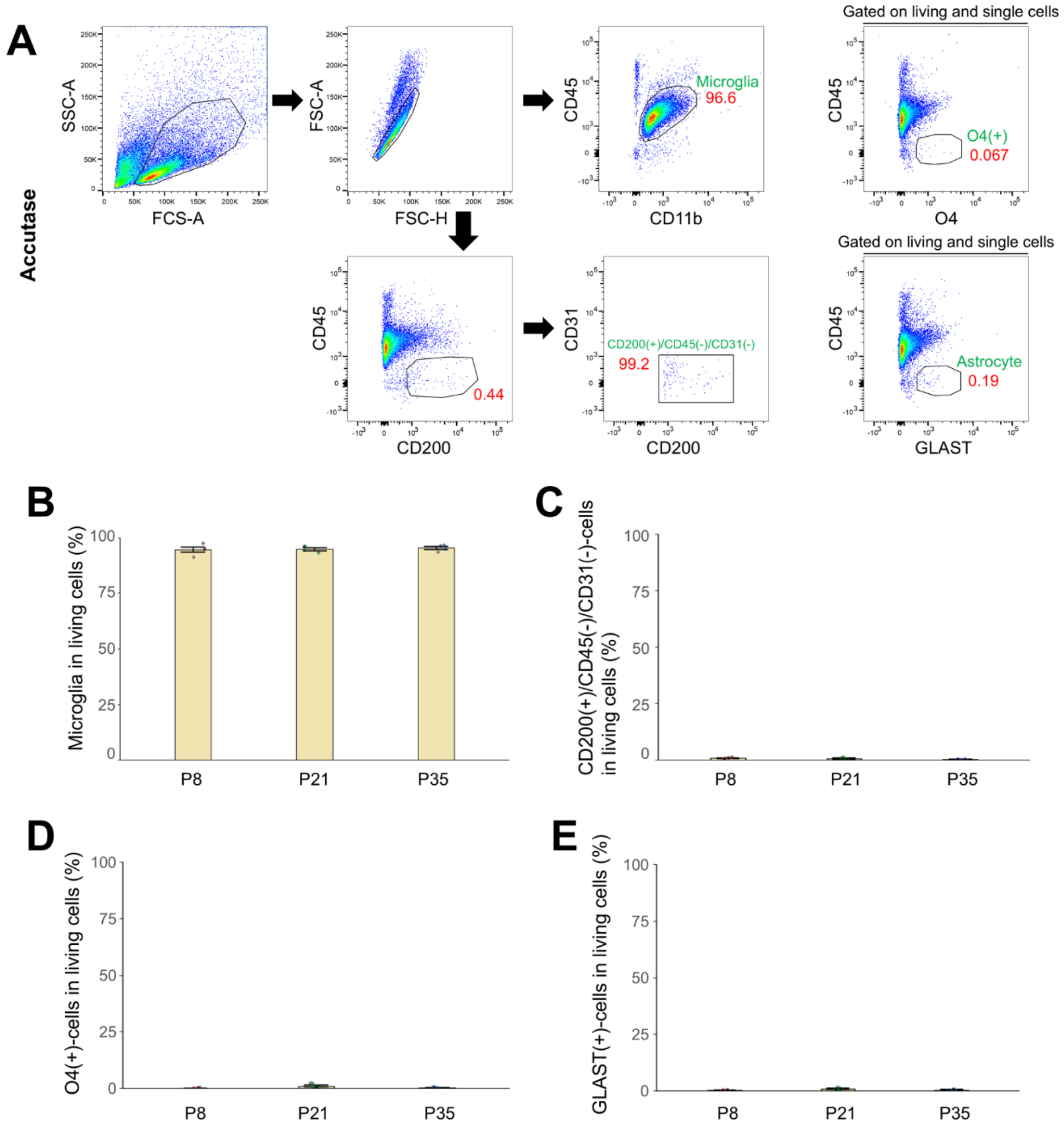

Fig. S3. Recovery rates of various brain cells after accutase treatment.

(A) Gating strategy for determining the yield of various brain cells after accutase treatment. The telencephalon of mice at P8 was used for flow cytometry. (**B-E**) Yields of microglia (**B**), CD200-positive neurons (**C**), O4-positive oligodendrocytes (**D**), and GLAST-positive astrocytes (**E**) after accutase treatment (n = 4 for each bar). Error bars represent mean ± SEM.
